# Supplementary material for: Low-Dose rIL-15 Protects from Nephrotoxic Serum Nephritis via CD8+ T Cells
Source: Cells. 2022 Nov 18;11(22):3656. doi: 10.3390/cells11223656 (PMC9688325; doi:10.3390/cells11223656)
Supplement: Supplementary file 1 [file cells-11-03656-s001.zip › cells-2005971-supplementary.pdf]

Low-dose rIL-15 protects from nephrotoxic serum nephritis via $CD8^+$ T cells

Agnes A. Mooslechner [1], Max Schuller [1], Katharina Artinger [1], Alexander H. Kirsch [1], Corinna Schabhüttl [1], Philipp Eller [2], Alexander R. Rosenkranz [1], Kathrin Eller [1,*]

[1] Division of Nephrology, Department of Internal Medicine, Medical University of Graz, 8036 Graz, Austria
[2] Intensive Care Unit, Department of Internal Medicine, Medical University of Graz, 8036 Graz, Austria
* Correspondence: kathrin.eller@medunigraz.at (KE)

Table of contents

Figure S1. Serum blood urea nitrogen, autologous antibody response, immune cell quantification in the spleen and peripheral blood in mice 7 days after NTS induction treated with low-dose rIL-15 or vehicle.

Figure S2. Quantification of CD8 memory subpopulations in the kidney, purity of transferred CD8 T cells, gene expression of T cell transcription factors in the kidney of mice 7 days after NTS induction treated with low-dose rIL-15 or vehicle. Autologous antibody response and iNKT cell quantification in kidney tissue in *CD8α*$^{-/-}$ mice 7 days after NTS induction and treated with or without rIL-15.

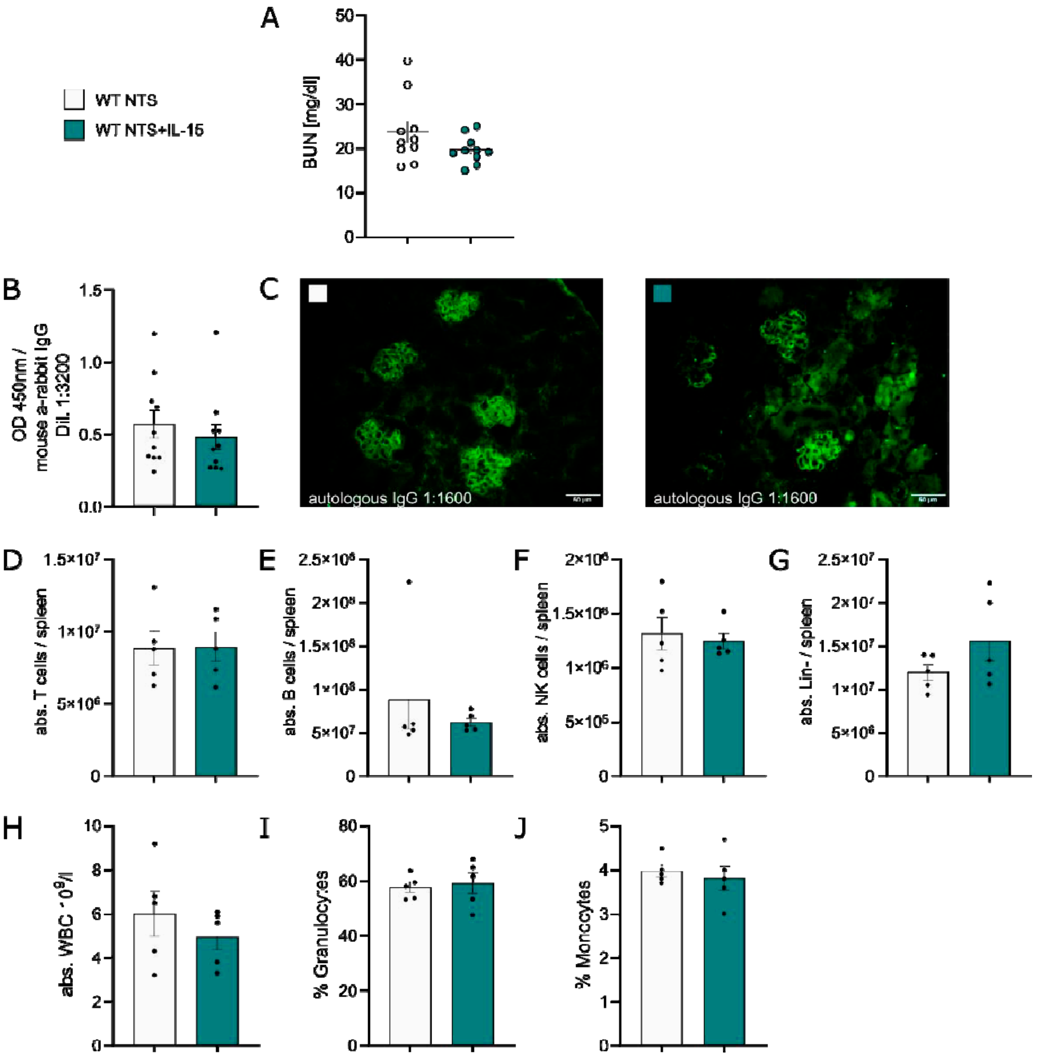

Figure S1. Serum blood urea nitrogen, autologous antibody response, immune cell quantification in the spleen and peripheral blood in mice 7 days after NTS induction treated with low-dose rIL-15 or vehicle. Data represent day 7 of NTS. **(A)** Quantification of blood urea nitrogen in serum. **(B)** Optical density of circulating mouse anti-rabbit IgG in serum and **(C)** representative staining of autologous IgG deposits in kidney tissue. Quantification of **(D)** CD45$^+$CD3$^+$CD90.2$^+$ T cells, **(E)** CD45$^+$CD19$^+$ B cells, **(F)** CD45$^+$NK1.1$^+$ NK cells, and **(G)** CD45$^+$CD19$^-$CD3$^-$CD90.2$^-$ NK1.1$^-$ Lineage negative cells in spleen. **(H)** White blood cell count and analysis of **(I)** granulocyte and **(J)** monocyte frequencies in peripheral blood. Statistical analysis used was Student's t-test or Mann-Whitney test. All data are mean ± SEM.

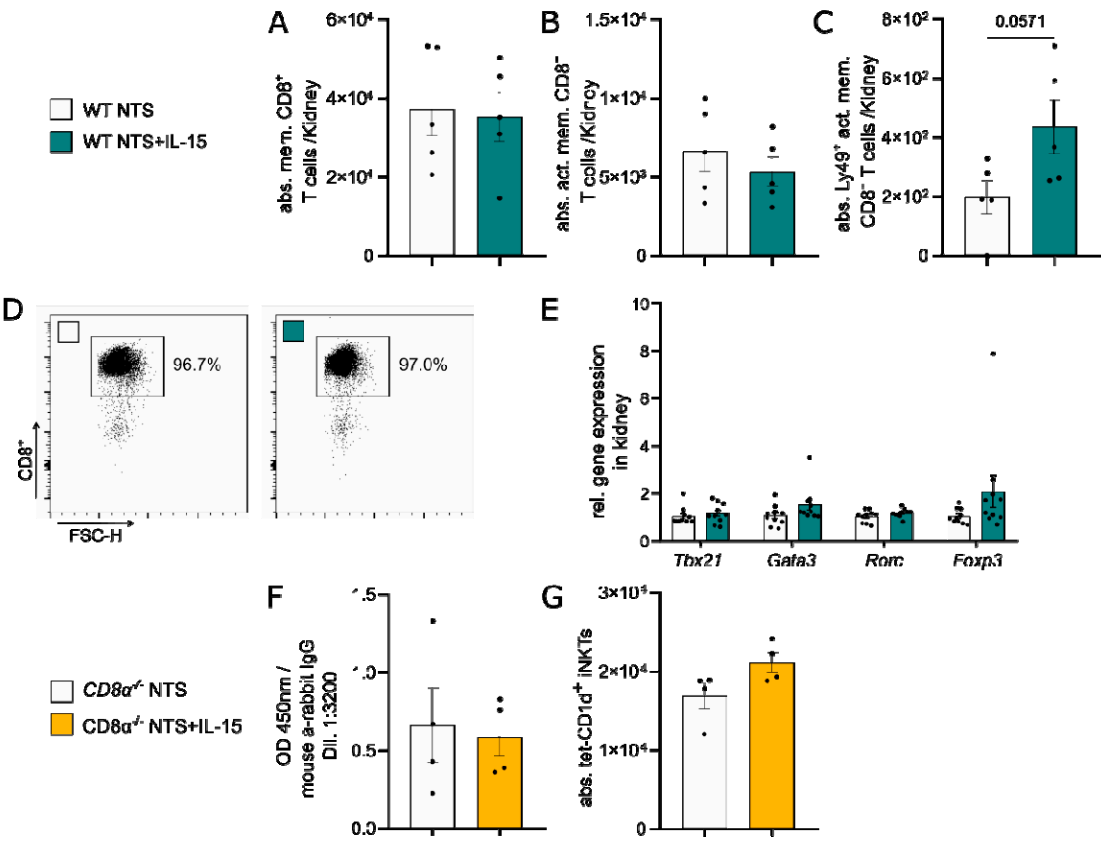

Figure S2. Quantification of CD8 memory subpopulations in the kidney, purity of transferred CD8 T cells, gene expression of T cell transcription factors in the kidney of mice 7 days after NTS induction treated with low-dose rIL-15 or vehicle. Autologous antibody response and iNKT cell quantification in kidney tissue in *CD8α*$^{-/-}$ mice 7 days after NTS induction and treated with or without rIL-15. Data represent day 7 of NTS. Quantification of **(A)** $CD44^+CD8^+$ memory T cells, **(B)** $CD122^+$ memory $CD8^+$ T cells, and **(C)** $Ly49^+CD122^+$ memory $CD8^+$ T cells in kidney tissue. **(D)** Dot plots showing purity of sorted $CD8\alpha^+$ cells from lymph nodes of NTS mice treated with control or IL-15, used for gene expression studies. Plots represent concatenated data of all samples per group. **(E)** Relative gene expression of *Tbx21*, *Gata3*, *Rorc*, and *Foxp3* in kidney tissue. **(F)** Optical density of circulating mouse anti-rabbit IgG in serum and quantification of **(G)** $CD45^+CD19^-CD3^-$tet-CD1d$^+$ iNKT cells in kidney tissue of *CD8α*$^{-/-}$ mice. Statistical analysis used was Student's t-test or Mann-Whitney test. All data are mean ± SEM.
